# A six-year risk assessment for dementia and Alzheimer's disease in the general population through immunoprecipitation-mass spectrometry plasma amyloid quantification

**DOI:** 10.1016/j.tjpad.2025.100186

**Published:** 2025-04-19

**Authors:** Germain U. Busto, Christophe Hirtz, Isabelle Carriere, Karim Bennys, Laure-Anne Gutierrez, Jana Kindermans, Catherine Helmer, Audrey Gabelle, Sylvain Lehmann, Claudine Berr

**Affiliations:** a INM, University of Montpellier, INSERM, 80 Av. Augustin Fliche, 34000, Montpellier, France; b Memory Resource and Research Center, Department of Neurology, University of Montpellier Hospital, 80 avenue Augustin Fliche, 34295, Montpellier, France; c University of Montpellier, IRMB, CHU Montpellier, 80 avenue Augustin Fliche, 34295, Montpellier, France; d University of Bordeaux, INSERM UMR U1219, Bordeaux Population Health (BPH) Research Centre, 146 rue Léo-Saignat. 33076, Bordeaux, France

**Keywords:** Population-based, Alzheimer’s disease, Dementia, Plasma, Amyloid

## Abstract

**Background:**

Identifying individuals at risk for dementia and Alzheimer’s disease (AD) in the general population (GP) is increasingly essential due to new diagnostic criteria and opportunities for effective interventions. Plasma-based biomarkers (pBB) offer a promising approach for detecting positive amyloid profile. However, their effectiveness in predicting clinical dementia and AD risk at the GP level remains largely unexplored.

**Objectives:**

To assess the risk of clinical dementia and AD using pBB amyloid biomarkers in GP using the most up-to-date proteomic techniques.

**Design:**

Case-cohort study randomly selected from a prospective cohort.

**Setting:**

The three-city community-living study.

**Participants:**

Over 65 years recruited from the electoral rolls of three French cities.

**Measurements:**

pBB amyloid levels (Aβ42, Aβ40 and APP669–711) were measured in the plasma using the mass spectrometry-based (IPMS)-Shimadzu modified technology. Patients were monitored for up to 6 years for incident dementia and AD according to DSM-IV and NINCDS/ADRDA criteria. Cox proportional hazard models adjusted for multiple covariables, including age and renal function, were used to estimate hazard ratios.

**Results:**

Plasma samples from 327 participants were analyzed with a mean age 83 years (80–87), 64.8 % females and a median follow-up time of 2.7 years (0.8–4.8) and including 121 incident dementia cases. Our findings indicate that the Aβ42/Aβ40 ratio, along with a composite score that encompasses APP669–711 and Aβ40/Aβ42 ratios, serves as significant predictors of clinical dementia [HR(95 %CI) = 3.52 (1.69–7.32), p-value<0.001 and 4.34 (2.06–9.17), p-value<0.001, respectively] and AD risk over a six-year period, while also accounting for age and sex interactions. Furthermore, elevated Aβ40 levels correlate with an increased risk of developing dementia (HR=2.56, 95 % CI 1.22–5.35, *p* = 0.01) and AD (HR=2.60, 95 %CI 1.06–6.36, *p* = 0.04), and our study confirms that Aβ42 concentrations are significantly influenced by renal function.

**Conclusions:**

This research advances the potential application of plasma amyloid biomarkers for assessing the risk of clinical dementia and AD in the general population within short period of time, positioning it as a valuable tool alongside existing plasma PT217 biomarkers or using ratio of both of them.

## 1 Introduction

Dementia and Alzheimer’s disease (AD) present an escalating public health challenge [1,2]. In light of recent advancements in diagnostic criteria for AD [[3], [4], [5]] and the potential for effective preventive and targeted therapeutic strategies [6,7], the identification of individuals at risk for dementia and AD within the general population (GP) and the predicting value of scalable biomarkers have become increasingly paramount [[6], [7], [8], [9], [10], [11], [12], [13]]. For some targeted therapies, the presence of amyloid positive status is mandatory [14,15]. Standard and validated amyloid biomarkers assessment in CSF and PET-brain imaging [[16], [17], [18], [19], [20], [21]] are complex to expand at a large scale detection level. Therefore, wide-scale screening of at-risk populations for dementia and AD using plasma-based biomarkers (pBB) emerges as a necessary strategy [22]. The pBB methods are not only accessible and easy to implement including in primary care [23] and with fully-automated quantification methods [24,25], but also cost-effective. With the recent development of innovative proteomics tools, the sensibility and specificity of amyloid plasma biomarkers have been considerably improved [26] and are now close to those of CSF and PET analyses [27]. To date, mass spectrometry-based (MS) techniques remain the best, in term of sensitivity and specificity, to diagnose AD among all tested amyloid assays [28,29]. Nakamura et *al.* notably established the excellent performances (AUC > 0.90) of a composite biomarker combining the APP669–711 (also known as Aβ−3–40)/Aβ42 and Aβ40/Aβ42 ratios to predict individual brain amyloid burden [30]. Moreover, several conditions (inflammation, renal dysfunction…) affect basal amyloid-β expression and might cause inter-individual variations, especially in a complex matrix as the plasma with very low concentrations of amyloid-β. Using Aβ42 in a ratio as Aβ42/APP669–711, improves its discriminative performance [31]. Expressing Aβ42 relative to two references, APP669–711 and Aβ40, both reflecting basal amyloid-β expression, combined in a composite score, exhibit even higher performances [30]. Our group later confirmed the relevance of amyloid plasma biomarkers, including MS-based amyloid biomarkers, relative to CSF, to diagnose early AD patients in memory clinic samples [32] or to predict CSF AT(N) profiles [33].

Most studies assessing the diagnostic accuracy and predictive value of amyloid plasma biomarkers are cross-sectional in design, often including patients exhibiting cognitive symptoms who are already entrenched in the neurodegenerative process; and/or employ stringent inclusion and exclusion criteria for participant selection, typically for randomized clinical trials testing disease-modifying therapies [29]. Such methodological limitations restrict the generalizability of their findings. To date, only a handful of studies have investigated the relationship between plasma amyloid biomarkers and dementia or AD within population-based cohorts [[34], [35], [36], [37], [38], [39]], and many of the earlier studies utilized outdated detection methods.

Collectively, these investigations suggest that lower baseline Aβ42/Aβ40 ratios are linked to an elevated risk of developing dementia or AD. However, the relevance of plasma amyloid biomarkers in real-world datasets, taking into account other cofounding factors such as renal function, remains to be determined. Furthermore, advancements in proteomic techniques hold promise for enhancing the accuracy of plasma amyloid biomarker detection, yet no studies to date have explored these associations among community-dwelling individuals using immunoprecipitation mass spectrometry (IP-MS).

In this study, we leverage the well-characterized Three-City Study (3C)—a robust French population-based cohort—to rigorously evaluate the six-year risk of progressing to dementia or AD associated with the most advanced quantification methods of plasma amyloid biomarkers. By elucidating the potential of these biomarkers, we aim to reinforce their critical role in early detection and risk stratification, ultimately contributing to more effective personalized management strategies for an optimal cognitive trajectory.

## 2 Methods

### 2.1 Study sample

The Three-city (3C) study is a community-living cohort of 9294 participants aged 65 years and over recruited between 1999 and 2001 from the electoral rolls of three French cities: Bordeaux, Dijon, and Montpellier. The baseline assessment and the follow-up visits included standardized questionnaires, clinical examination, and detailed cognitive evaluations allowing active dementia screening [40]. Protocol was approved by the Ethics Committee of the Hospital of Kremlin-Bicêtre and Sud-Méditerranée III, and each participant signed an informed consent.

**Fig. 1:**
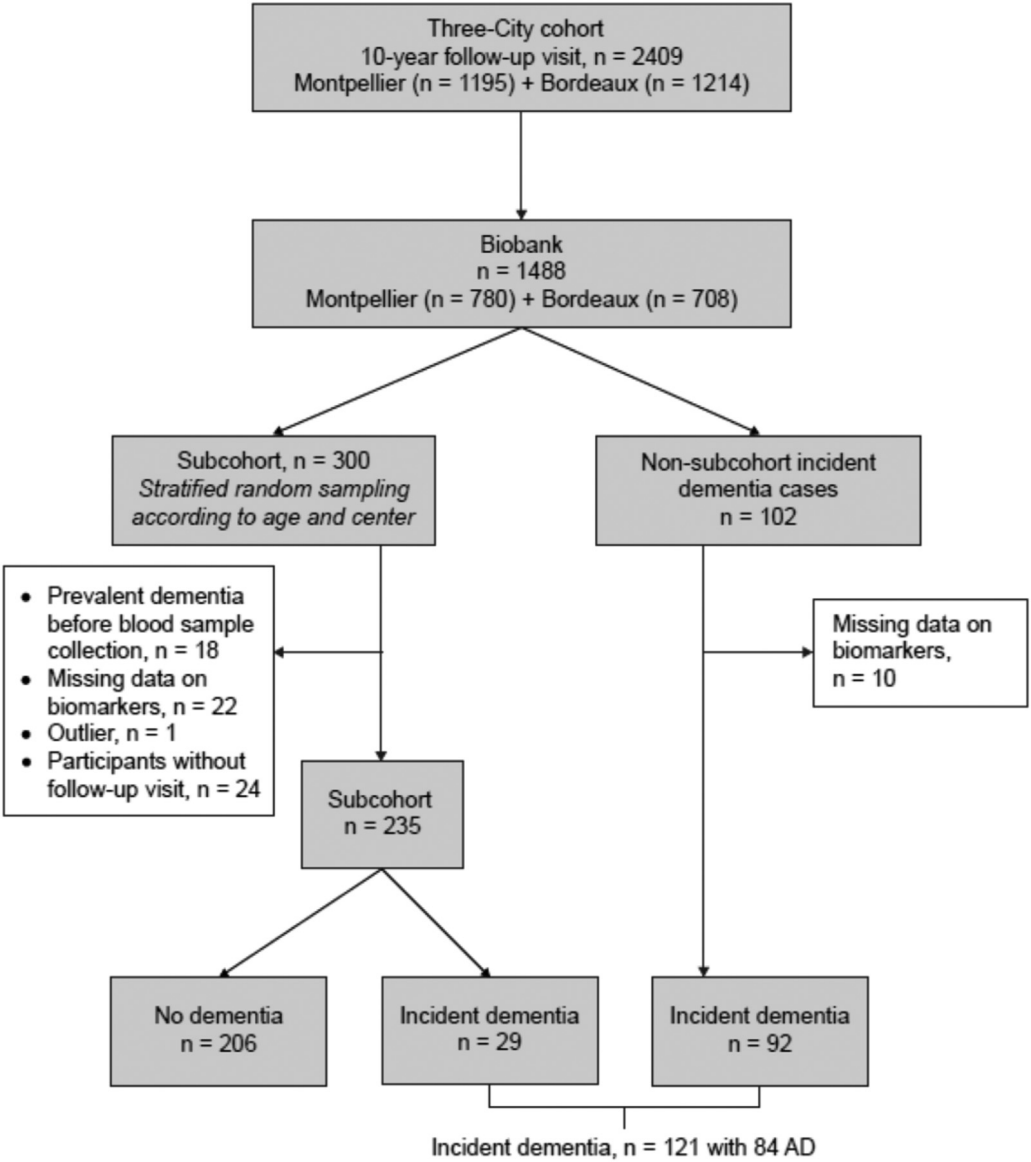
Flowchart of the study design: association between plasma amyloid biomarkers and all-cause dementia risk in the Three-City cohort.

Among the 1214 participants from Bordeaux and 1195 participants from Montpellier who completed the 10-year follow-up visit - established as the baseline for the present study - blood samples were collected from volunteers (*n* = 1488, aged over 75) with aliquots of plasma stored (Fig. 1). Participants were subsequently monitored for up to 6 years following blood sampling.

Individuals with plasma Aβ-peptide quantifications (*n* = 327) were all originating from the 3C study blood biobank constituted at the 10-year follow-up (*n* = 1488) (Fig. 1). IP-MS quantifications being economically limited, a case-cohort sampling method [41] combining two selection pathways for inclusions was used: [1] a subcohort of 300 individuals randomly selected taking 1/5th of the individuals from the blood biobank thus including cases and non-cases; [2] all remaining non-subcohort incident dementia cases from the blood biobank (*n* = 102 before exclusions) (Fig. 1). The newly generated case-cohort gathered individuals from pathways 1 and 2.

As we combined selection pathways, individuals had either been sampled or not sampled thus with unequal contributions throughout time. To account for this differential contribution, subjects originating from pathway 1, somehow “under-weighted”, were affected a weight of 5 to compensate for the sampling rate as recommended for case-cohort [41].

From the blood biobank, the sub-cohort (pathway 1, *n* = 300, sampling rate of 1 in 5) was randomly selected stratifying by study center and age (grouped by 5-year intervals). Additionally (pathway 2), 102 dementia cases diagnosed during the 6-year follow-up period, with available plasma samples available, were included, yielding a total of 402 participants for the current case-cohort study [41].

Following the exclusion of 18 dementia cases diagnosed prior to blood sampling, 32 participants with control samples that were out of range (due to missing data), one participant with outlier values for Aβ40 and Aβ42 (less than mean - 3SD) and 24 without clinical assessment after the 10-year follow-up, a sub-cohort of 235 participants was established (pathway 1). Of these, 206 were classified as no dementia cases and 29 as incident dementia cases. Additionally, we selected 102 incident dementia cases, and after excluding 10 due to missing plasma biomarkers data, 92 incident dementia cases were ultimately analyzed (pathway 2). Thus, the final analyzed sample consisted of 327 participants: 206 sub-cohort non-dementia cases, 29 sub-cohort incident dementia cases (pathway 1) and 92 non-sub-cohort incident dementia cases (pathway 2). Notably, among the 121 incident dementia cases, 84 (69.4 %) were diagnosed as probable or possible AD (Fig. 1).

### 2.2 Dementia diagnosis

At the time of enrolment in the cohort and during each follow-up visit, all recruited participants underwent an extensive cognitive and functional evaluation conducted by a neuropsychologist. Participants exhibiting signs of possible decline were subsequently examined by a neurologist. The final diagnostic determination was achieved through a case review by an independent committee of neurologists who reached a consensus on the diagnosis based on the DSM-IV criteria [42]. AD was classified according to the National Institute of Neurological and Communicative Disorders and Stroke and the Alzheimer’s Disease and Related Disorders Association (NINCDS/ADRDA) criteria [43]. The date of dementia diagnosis was defined as the midpoint between the last follow-up visit at which no dementia was identified and the first follow-up visit where dementia was confirmed.

### 2.3 Plasma samples and amyloid detection

K2-EDTA blood samples were obtained through venipuncture. After a 15-minute centrifugation at 1500x*g* within four hours from collection, plasma was divided into 0.5 mL-aliquots in 1.5–2 mL polypropylene tubes (Sarstedt, Germany) and stored at −80 °C until biochemical assessment.

As reported previously [32], the IPMS-Shimadzu technology was slightly modified from Nakamura et al. [30]. Plasma Aβ levels, including Aβ42, Aβ40 and APP669–711, were measured using a linear MALDI-TOF mass spectrometer (AXIMA Assurance, Shimadzu) after being purified by immunoprecipitation (IP-MS).

The IP-MS method involved two consecutive IP steps through magnetic beads (DynabeadsTM M-270 Epoxy) coated with mouse monoclonal anti-Aβ antibodies. For this, 250 µL of plasma EDTA were mixed with an equal volume of internal standard Aβ1–38 and containing 0.2 % w/v n-dodecyl-β-d-maltoside (DDM), 0.2 % w/v n-nonyl-β-d-thiomaltoside (NTM) and 800 mM N-Acetylglucosamine (GlcNAc). This was prepared 30 min before starting the automated IP procedure.

After the second IP and elution, samples were spotted on four wells of a MALDI plate which already contained a dried prespotted matrix of 0.5 mg/mL α-cyano-4-hydroxycinnamic acid (CHCA), 0.2 % methanediphosphonic acid (MDPNA), 70 % acetonitrile (ACN) and 0.05 % trifluoroacetic acid (TFA). Samples were analyzed using a 337-nm nitrogen laser in the positive ion mode [30].

Aβ42 was then expressed relative to APP669–711 (Aβ−3–40) and Aβ40, both reflecting basal amyloid-β expression level. The IPMS composite biomarker was generated by averaging the standardized scores of APP669–711/Aβ42 and Aβ40/Aβ42 ratios [30].

### 2.4 Baseline covariates

Baseline covariates were assessed at inclusion or at the time of blood sampling. Socio-demographic variables included sex, age, study center, years of education, and living arrangement (living alone or not). Health-related covariates encompassed hypertension (blood pressure ≥140/90 mmHg or treated or declared), diabetes (either treated or declared) and self-reported history of cardiovascular diseases (CVD) (stroke, angina pectoris, myocardial infarction, cardiac and vascular surgery and heart rhythm disorder). Body mass index (BMI, weight/height^2^) was categorized in four categories: underweight (<20), normal [[20], [21], [22], [23], [24], [25]], overweight [[25], [26], [27], [28], [29], [30]] and obese (≥30). Participants with at least one ε4 allele of the APOE gene were defined as APOE ε4 carriers. The estimated glomerular filtration rate (eGFR) was calculated with the CKD Epidemiology Collaboration formula [44,45].

### 2.5 Statistical analysis

Weighted Cox proportional hazards regression with the robust variance and counting process approach was used to estimate the hazard ratios (HRs) and 95 % confidence intervals (CI) for the association between each biomarker and dementia risk over the 6-year period. Variance was estimated using the sandwich variance estimate. Time in the study was used as the time scale so age could be added as a covariate. For individuals in the sub-cohort, follow-up started from the date of blood draw until the diagnosis of dementia or censoring.

Sub-cohort non-cases were weighted by 5 (inverse sampling rate) until censoring. Sub-cohort cases were weighted by 5 until just before dementia and by 1 at the dementia diagnosis time. Cases not included in the sub-cohort were not considered at risk until just before dementia diagnosis, at which point they were assigned a weight of 1 for the moment of diagnosis [46].

Because of non-linear associations with dementia risk, biomarkers were analyzed as tertiles with the lowest tertile as the reference for Aβ40 and the composite biomarker and the highest for Aβ42 and the Aβ42/Aβ40 ratio. Curves of dementia-free probability stratified by biomarker tertiles were plotted using the Breslow's method of estimation due to the counting process approach. Models were firstly adjusted for age, age^2^, study center, educational level and gender (model 1). The model 2 was further adjusted for BMI, eGFR, diabetes, cardiovascular pathologies, hypertension, living alone and APOE4 genotype and used multiple imputations for missing covariates. These covariates were selected on the basis of previous studies [[34], [35], [36], [37], [38], [39]]. We used 10-fold multiple imputations by chained equations for missing data on participant covariates (BMI, diabetes, APOE and eGFR). The percentage of missing values ranged from 0.6 % to 5.2 %. The weighted Schoenfeld residuals were used to verify the proportional hazards assumption that was met for all models.

Additional analyses were performed to assess the robustness of the results in the AD subtype only. Sub-cohort non-AD cases were censored while non-AD cases that were not in the sub-cohort were excluded. As the plasma Aβ40 and Aβ42 were demonstrated to be higher in patients with impaired renal function [47,48] we also studied the associations with all-type dementia risk in the group of participants with normal eGFR (≥60 mL/min/1.73m^2^). Interactions with sex and age (median cut-off 82.6 years) were tested for all-type dementia risk and the results presented as forest plots.

All the analyses were carried out using SAS, version 9.4 (SAS Institute, Cary, NC).

## 3 Results

### 3.1 Baseline characteristics

Baseline demographics, clinical characteristics, and plasma biomarkers profiles of the 327 participants are summarized in Table 1. Overall, the median baseline age [interquartile range (IQR)] was 82.6 (80.0–86.5) years, 64.8 % were women and the median follow-up time was 2.7 (IQR 0.8–4.8) years. The median follow-up time was 4.6 (IQR 2.5 - 5.4) years for the 206 participants censored without dementia and 1.6 (IQR 0.7 - 2.2) years for the 121 participants with incident dementia. As anticipated, those with incident dementia were significantly older, less educated, exhibited greater cognitive impairment, and presented with higher baseline levels of plasma Aβ40 and composite biomarkers, conversely, the Aβ42/Aβ40 ratio was lower. Even if not significant, the incident dementia cases appeared more likely to be women, living alone, APOE4 carriers and had a greater likelihood of diabetes and cardiovascular conditions.

**Table 1:** Baseline characteristics (*N* = 327).

		**Non-cases (*n*=206)**	**Dementia cases (*n*=121)**	***P-value***
	n	**n ( %)**	**n ( %)**	
Gender				0.12
Male	115	79 (38.35)	36 (29.75)	
Female	212	127 (61.65)	85 (70.25)	
Living alone				0.12
No	191	127 (61.65)	64 (52.89)	
Yes	136	79 (38.35)	57 (47.11)	
ApoE (ε4 ( or +/+)				0.50
Non carrier	253	163 (82.74)	90 (79.65)	
Carrier	57	34 (17.26)	23 (20.35)	
Education				**0.007**
< 6 years	79	39 (18.93)	40 (33.06)	
6–11 years	87	54 (26.21)	33 (27.27)	
>11 years	161	113 (54.85)	48 (39.67)	
Hypertension				0.51
No	72	43 (20.87)	29 (23.97)	
Yes	255	163 (79.13)	92 (76.03)	
Body mass index				0.10
Underweight (<20)	24	14 (6.93)	10 (8.70)	
Normal [20–25]	142	84 (41.58)	58 (50.43)	
Overweight [25–30]	122	80 (39.60)	42 (36.52)	
Obese (≥30)	29	24 (11.88)	5 (4.35)	
Diabetes				0.17
No	287	184 (90.20)	103 (85.12)	
Yes	38	20 (9.80)	18 (14.88)	
Cardiovascular pathologies				0.68
No	258	164 (79.61)	94 (77.69)	
Yes	69	42 (20.39)	27 (22.31)	
eGFR (mL/min/1.73m2)				0.80
<45	29	19 (9.31)	10 (8.33)	
45–60	86	52 (25.49)	34 (28.33)	
60–90	203	130 (63.73)	73 (60.83)	
≥90	6	3 (1.47)	3 (2.50)	

	N	Median (IQR)	Median(IQR)	

Age (years)	327	81.7 (79.1;85.7)	84.6 (81.3;87.6)	**<0.0001**
MMSE, /30	322	29 (28;29)	27 (25;29)	**<0.0001**
eGFR (mL/min/1.73m2)	324	68.8 (56.4;78.6)	67.5 (53.2;78.1)	0.86
Aβ40, pg/mL	327	8.39 (7.08;9.93)	9.06 (7.78;10.47)	**0.02**
Aβ42, pg/mL	327	0.37 (0.31;0.45)	0.37 (0.32;0.42)	0.77
Composite biomarker	327	0.21 (−0.43;0.63)	0.51 (−0.01;1.05)	**<0.0001**
Aβ42/Aβ40 ratio	327	0.044 (0.039;0.049)	0.040 (0.037;0.046)	**0.001**

Hypertension is defined as BP ≥140/90 mmHg or treated or declared, diabetes as treated or declared and depression as treated or CES-*D* > 16 for men and >22 for women. Cardiovascular pathologies include stroke, angina pectoris, myocardial infarction, cardiovascular surgery and heart rhythm disorder. The estimated glomerular filtration rate (eGFR) was calculated with the CKD Epidemiology Collaboration formula. The composite biomarker was generated by averaging the standardized scores of APP669–711/Aβ42 and Aβ40/Aβ42 ratios. Chi-2 test was used for qualitative variables while Wilcoxon test for the quantitative ones. MMSE: Mini Mental State Examination.

### 3.2 Dementia and AD risk stratification based on plasma biomarkers

**Fig. 2:**
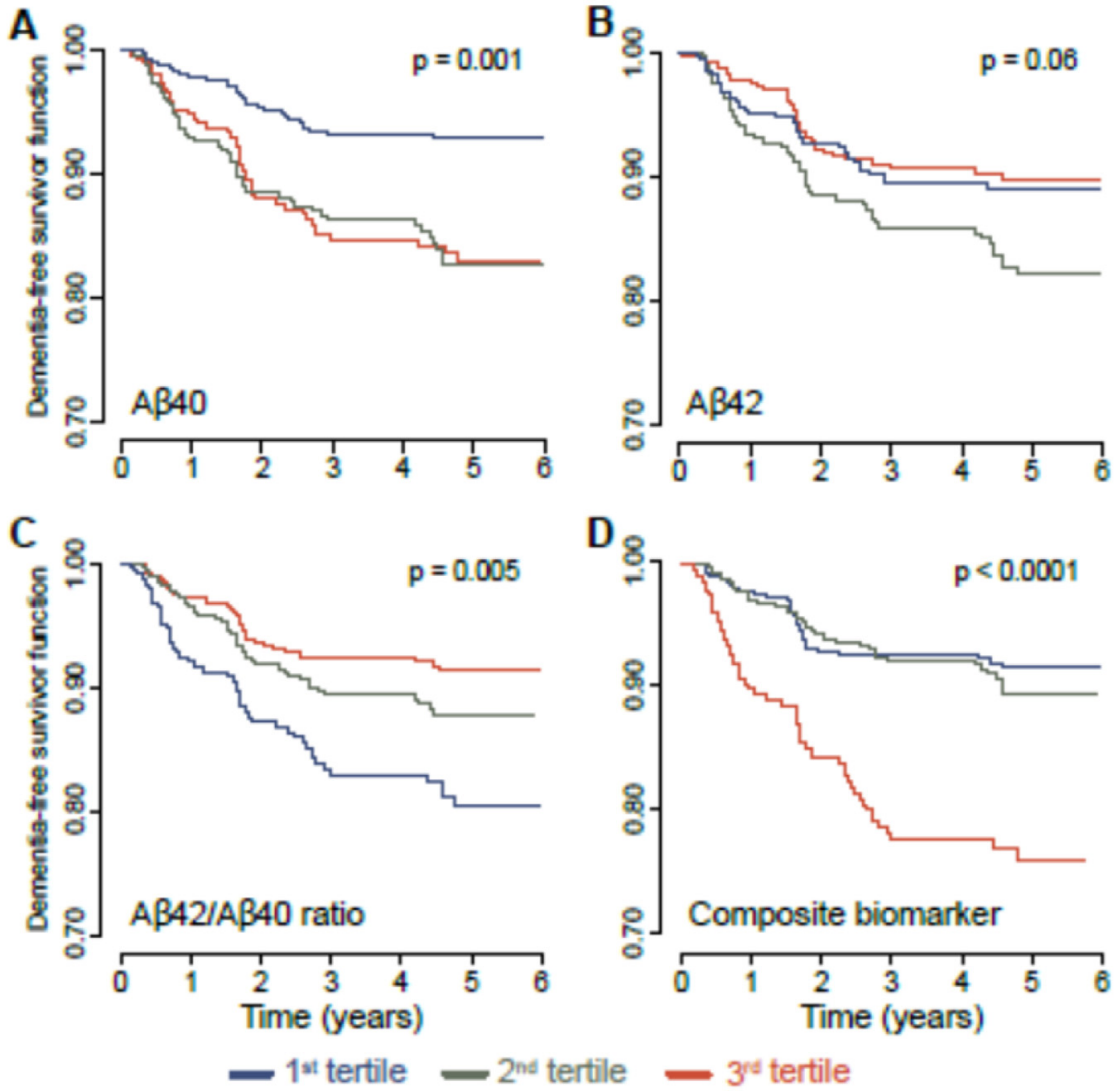
Dementia-free survival probability during follow-up according to tertiles of plasma biomarkers measured using IP-MS at baseline, *n* = 327. Dementia-free survival probability during follow-up in the 3C cohort according to Aβ40 concentration tertiles (Fig. 2A), to Aβ42 (Fig. 2B) to the Aβ42/Aβ40 ratio (Fig. 2C) or to the amyloid composite biomarker (Fig. 2D). The Breslow's method of estimation was used to account for the counting process approach. The curves are non-adjusted for the covariates.

Fig. 2 displays the non-adjusted risk curves for dementia-free survival probability during the 6-year follow-up, stratified by tertiles of plasma Aβ42, Aβ40, Aβ42/Aβ40 ratio and amyloid composite biomarkers. Individuals at the highest risk for developing dementia were those in the highest tertile for the composite biomarker (p-value<0.001) and the lowest tertile for the Aβ42/Aβ40 ratio (p-value=0.005). Then, participants with median and highest tertiles of Aβ40 (p-value=0.001) or median tertile for Aβ42 (p-value=0.06). Importantly, these findings remained significant after adjusting for multiple covariates, including, age, study center, gender, education, BMI, estimated GFR rate, diabetes, cardiovascular pathologies, hypertension, living alone and ApoE4 genotype (Table 2). Participants in the highest tertile of the composite biomarker and the lowest tertile of Aβ42/Aβ40 ratio remained at higher dementia risk [HR(95 %CI) = 4.34 (2.06–9.17) and 3.52 (1.69–7.32), respectively]; and in less magnitude of dementia risk for participants in the highest and the median tertile of Aβ40 [HR(95 %CI) = 2.56 (1.22–5.35) and 2.38 (1.20- 4.69), respectively] and the median tertile of Aβ42 [HR(95 %CI) = 2.20 (1.13–4.27)]. Additionally, these results were similar when the outcome was restricted to AD cases only (Table S1, 84 CEAD cases/300) except for Aβ42 for which no significant difference was found.

**Table 2:** Association of plasma biomarkers with all-type dementia risk (*N* = 327).

			Model 1		Model 2	
Biomarkers		N	HR [95 %CI]	*P-value*	HR [95 %CI]	*P-value*
Aβ40 (pg/mL)	T1: ≤7.76	108	1		1	
	T2:]7.76–9.48]	110	**2.25 [1.26–4.05]**	**0.007**	**2.38 [1.20–4.69]**	**0.01**
	T3: >9.48	109	1.78 [0.95–3.32]	0.07	**2.56 [1.22–5.35]**	**0.01**
Aβ42 (pg/mL)	T1: ≤ 0.33	109	1.71 [0.90–3.25]	0.10	1.73 [0.80–3.75]	0.16
	T2:]0.33–0.41]	109	**2.08 [1.17–3.72]**	**0.01**	**2.20 [1.13–4.27]**	**0.02**
	T3:>0.41	109	1		1	
Composite biomarker	T1: ≤ −0.009	109	1		1	
	T2:]−0.009–0.62]	109	1.33 [0.74–2.40]	0.35	1.62 [0.81–3.22]	0.17
	T3: >0.62	109	**3.50 [1.85–6.62]**	**0.0001**	**4.34 [2.06–9.17]**	**0.0001**
Aβ42/Aβ40 Ratio	T1: ≤ 0.039	109	**2.42 [1.33–4.39]**	**0.004**	**3.52 [1.69–7.32]**	**0.0008**
	T2:]0.039–0.045]	108	**2.02 [1.06–3.87]**	**0.03**	2.00 [0.94–4.25]	0.07
	T3: >0.045	110	1		1	

The reference is the lowest tertile for Aβ40 and composite biomarker and the highest tertile for Aβ42 and Aβ42/Aβ40 ratio.

Model 1 adjusted for age, age^2^, center, gender and education.

Model 2: Model 1 further adjusted for BMI, estimated glomerular filtration rate, diabetes, cardiovascular pathologies, hypertension, living alone and ApoE4 genotype and with multiple imputations for missing covariates.

HR= Hazard Ratios.

As plasma amyloid may be influenced by age and renal function, we further restricted the analyses to participants with optimal eGFR (Table S2, *n* = 209) to identify any potential effect of this factor. The results remained significant for the same tertiles of the composite biomarker and only for the median tertile of Aβ40. In addition to the lowest tertile, the median one of Aβ42/Aβ40 ratio was also associated with an increased risk of dementia. Moreover, the median and lowest tertiles of Aβ42 were both associated with a higher dementia risk when compared to the highest tertile.

**Fig. 3:**
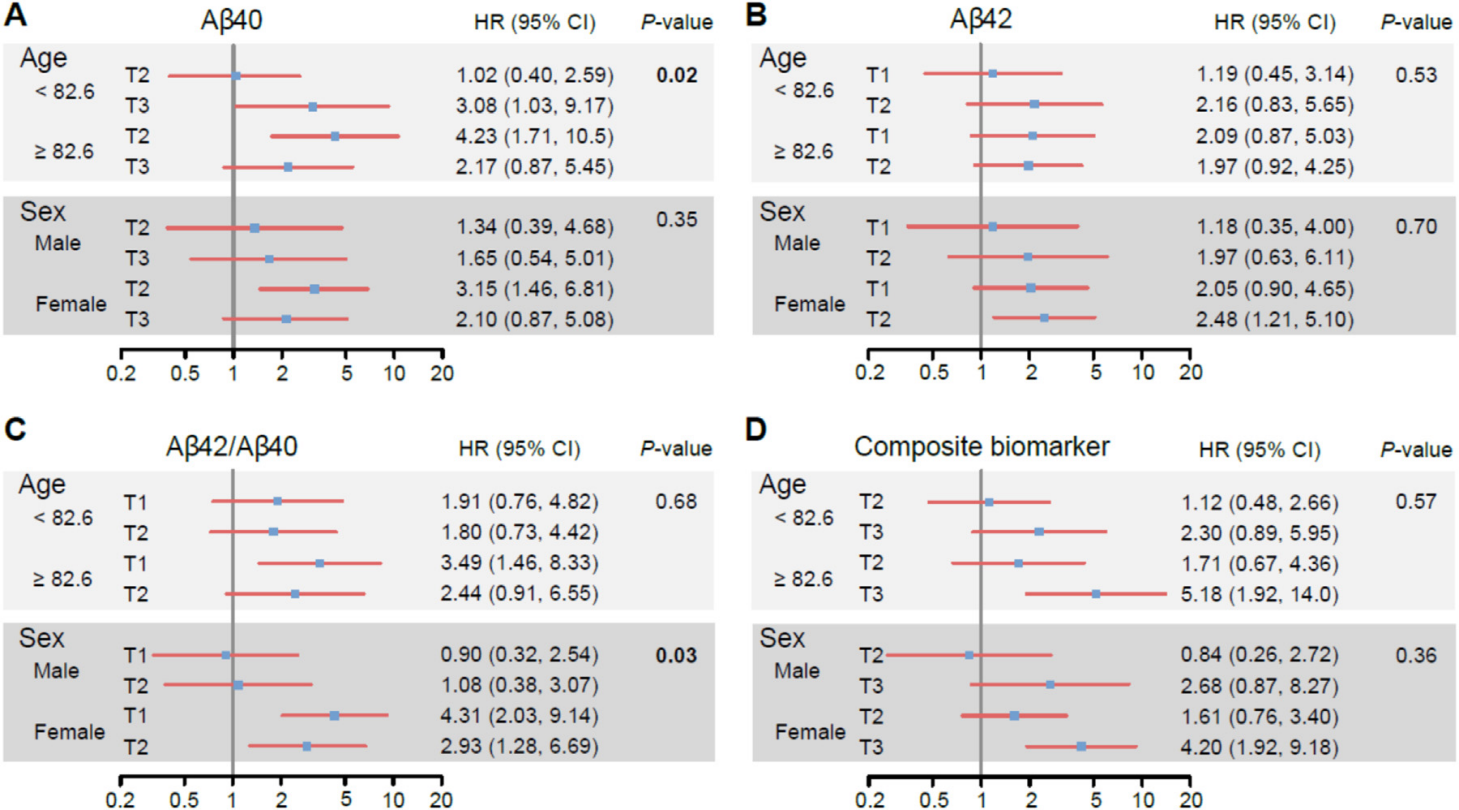
Association of plasma biomarkers measured using IP-MS at baseline with all-type dementia risk stratified on age and gender, *n* = 327. Dementia/AD hazard ratios associated with Aβ40 tertiles, relative to the lowest tertile, and stratified according to age or sex (Fig. 3A). Hazard ratios associated with Aβ42 tertiles relative to the highest tertile (Fig. 3B). Hazard ratios associated with Aβ42/Aβ40 lowest tertiles (Fig. 3C). Hazard ratios associated with the highest tertiles of the composite amyloid biomarker (Fig. 3D). Hazard ratio (HR) and confidence interval (CI, log scale) were adjusted for age, sex (if applicable), education and eGFR. P-values are associated with the test of interaction between a biomarker and each subgroup.

Finally, exploratory stratified analyses revealed that the association of Aβ40 levels and the risk of incident overall dementia was particularly pronounced among the oldest participants, specifically those aged over 82.6 years at baseline (p-value for interaction= 0.02). In contrast, the association of Aβ42/Aβ40 ratio and incident dementia was more apparent in women (p-value for interaction=0.03) (Fig. 3). No other formal interaction terms were found to achieve statistical significance.

## 4 Discussion

Using, to date, the most advances and performant proteomic platforms for plasma biomarkers quantification, i.e. IPMS, we demonstrated that plasma amyloid concentrations measured in a population-based cohort serve as indicators of increased risk for clinical dementia and AD years prior to diagnosis. Our findings revealed that elevated levels of Aβ40 are associated to a higher risk of all-cause dementia, especially among the oldest participants, and emphasized the importance of considering renal function when assessing the dementia risks associated with Aβ42 quantification. Furthermore, we confirmed that the Aβ40/Aβ42 ratio is significantly associated with incidence of all-cause dementia taking into account potential confounders, including eGFR, which has been considered only in a few previous studies [35,37]. Similar conclusions may be drawn through the use of an amyloid composite score combining two ratios, APP669–711/Aβ42 and Aβ40/Aβ42.

Aβ42 and Aβ40 had been the most extensively studied biomarkers in the plasma; however, initial findings regarding their reliability were inconsistent [49,50]. The measurement of AD-related Aβ in the plasma is influenced by numerous factors including peripheral production, degradation and binding to peripheral proteins [50]. Moreover, these AD biomarkers are present in significantly lower concentration in plasma compared to CSF necessitating the use of highly sensitive techniques as Simoa or IPMS [28]. Furthermore, standardized operating procedures for pre-analytical sample handling are critical to ensure accurate and reproducible results [51].

In the present study, we disclosed that elevated level of Aβ40 concentrations were linked to a higher risk for all-cause dementia including AD. Our results are consistent with at least two studies, one prospective case-cohort from the Rotterdam study and a previous sub-cohort from the 3C study both showing that high Aβ40 concentrations are associated with increased risk of dementia [35,36]. Interestingly, high Aβ40 in the CSF has been previously associated with AD [52]. However, a meta-analysis of seven population-based studies indicated no association between blood Aβ40 and dementia/AD risk [37]. According to the authors, differences in blood sampling, storage protocols, assays, sample size, follow-up time and analysis strategies explained the important heterogeneity between studies. The present study, employing state-of-the-art assays and consensual operating procedures, aims to provide additional insights strengthening the association between high plasma Aβ40 concentrations and dementia risk.

Prospective population-based studies quantifying plasma Aβ42 were equally puzzling with, for example, Mayeux et al. showing increased dementia risk with high concentration [34] while van Oijen et al. did not find association [35] and others disclosed that low Aβ42 was associated with dementia [38,39]. In Chrouraki et al., meta-analyzed data indicated that lower plasma Aβ42 was significantly associated with incident dementia [37]. In our case, only the intermediate tertile of Aβ42 was associated with an increased risk of dementia, and the *p*-value close to significance for AD, most likely reflecting a lack of power. As mentioned above, plasma amyloid is influenced by numerous factors including age and renal function which confounding effect might however be overcome using the Aβ42/Aβ40 ratio [47]. While our model was adjusted for those parameters, we speculate that complex interactions between multiple parameters might somehow hide the association. Interestingly, if participants with the optimal renal function (eGFR ≥60 ml/min/1.73m²) were considered (Table S2), the lowest Aβ42 concentrations were predictive of dementia thus allowing to reconcile with current consensus. Taken together with recent data indicating influence of renal function on β-amyloid [47,48], our results strongly argue for an adjustment with renal function, especially in aged populations, and maybe prefer the Aβ42/Aβ40 ratio to simplify interpretation.

Lower plasma Aβ42/Aβ40 ratio had almost constantly been shown to be predictive or associated with dementia and AD either in prospective study [37] or with cross-sectional approaches [53]. In our case, the lowest Aβ42/Aβ40 ratio was associated with a 3.5 to 2.5-time increased risk in developing clinical dementia or AD. Those results are consistent with previous associations obtained in the 3C cohort, the Rotterdam study or the Framingham Heart Study [[35], [36], [37],^39^]. Interestingly, all studies had similar results, even if they used different quantification assays (ELISA, xMAP, Simoa, IPMS), follow-up times (5 to 15 years), sample sizes (458 to 4444) or analysis strategies. Moreover, in our case, significance of the HR was consistent across models thus making Aβ42/Aβ40 ratio measured with IPMS particularly attractive for evaluation in GP. Eventually, one could reconcile Aβ40 and Aβ42 respective participation by using the ratio that combines individual effects to generate a synergistic effect [35,47]. Our study confirms, in GP, that the Aβ42/Aβ40 ratio remains, among amyloid biomarkers, a robust tool, if not the best, to predict evolution toward clinical AD. This is particularly relevant within the ongoing debate surrounding AD criteria, specifically concerning how the community categorizes individuals who are cognitively unimpaired yet exhibit amyloid-positive markers—as either having AD or being at risk for AD. This situation highlights the necessity of establishing a clear clinical progression over time for individuals with amyloid-positive biomarkers within a population-based cohort. Our findings suggest that amyloid pBB, measured using recent proteomic techniques, may help identify the most appropriate targeted participants for intervention. The heterogeneity in cognitive trajectories among individuals can be attributed to various factors, including recent insights into proteomic profiles [54] and the amyloid-predominant AD neuropathological change (AP-ADNC) [55]. To promote personalized medicine and effectively identify treatment responders, validated biomarkers must be established and utilized. The plasma-based Aβ42/Aβ40 ratio could serve as a valuable complement to plasma PT217 biomarkers in this endeavor.

Notably, females with low Aβ42/Aβ40 ratio at baseline were at a greater risk of all-cause dementia than males, consistent with results obtained with p-tau181 in the plasma, indicating that elevated values were associated with higher odds of AD dementia in females [56]. As women are disproportionally affected by AD, our results strengthen the importance of considering sexual dimorphism and the necessity of patient stratification for disease risk assessment, diagnosis and eventually treatment [57]. Aβ42/Aβ40 ratio assessment using IPMS could thus reveal a useful tool toward that goal as it was able to capture a sex difference in dementia risk.

The composite amyloid biomarker, combining z-scores of the APP669–711/Aβ42 and Aβ40/Aβ42 ratios, provides conclusions that align closely with those derived from the Aβ42/Aβ40 ratio. The highest composite score values were associated with increased risk of dementia/ AD across all model considered. Notably, this composite biomarker is unique in that it does not exhibit a correlation with renal function (Table S3) suggesting that expressing pathological Aβ42 in relation to two amyloid references effectively mitigates the influence of confounding factors, at least renal function [30]. Our results are consistent with Lim YY et *al.* showing that the Aβ status, determined using the composite amyloid biomarker, is also associated with episodic memory and executive function declines, two hallmarks of AD [58].

The limits of the study include the relatively small number of samples available for quantification and the follow-up length. This would not allow us to evaluate the impact of APOE4 either. It is also noteworthy that all participants were over the age of 75 at time of blood sampling. The most recent recommendations for AD diagnosis, at least for research, rely on amyloid biomarkers measured through CSF or PET-amyloid [4] limiting the biological relevance of AD diagnosis in the present study. However, for the time being, a clinically-based diagnosis remains more compatible with large scale population-based study. Other fluid biomarkers, as PT217, recently recommended as a proxy for brain amyloidopathy [4] would have been of great interest, especially to assess amyloidopathy at baseline and compare with our IP-MS results.

However, our study possesses some strength including the rigorous three-steps processes employed for accurate AD diagnosis, with a complete set of neuropsychological evaluation. Furthermore, we were able to use state-of-the-art proteomic technique to measure amyloid on sample derived from GP. Importantly, we incorporated the most recent findings demonstrating that renal function significantly influences plasma amyloid concentrations.

Our findings support the utility of blood-based biomarkers as effective predictors of dementia in general aged population when assessed with the latest proteomic technic. We propose that Shimadzu immunoprecipitation mass spectrometry-IPMS could be adopted for large-scale implementation by focusing solely on the Aβ42/Aβ40 ratio, thereby reducing costs by approximately one-third compared to the composite score. Our results could be strengthened by complementary analysis in subjects under age 75.

## Abbreviations

Aβ40, 40-amino acid-long amyloid beta peptide; Aβ42, 42-amino acid-long amyloid beta peptide; AD, Alzheimer’s disease; AUC: area under the curve; CI, confidence interval; CSF, cerebrospinal fluid; eGRF, estimated glomerular filtration rate; HR, hazard ratio; IP, immunoprecipitation; IQR, interquartile interval; LP, lumbar puncture; MS: mass spectrometry; PET, positron emission tomography.

## Declaration of generative AI and AI-assisted technologies in the writing process

None were used in the writing process.

## Funding

This work was funded, for its realization, by a grant from “France Alzheimer” and support from the “Fondation pour la Recherche Médicale” (FRM).

## CRediT authorship contribution statement

**Germain U. Busto:** Writing – original draft, Visualization, Methodology, Investigation, Formal analysis, Data curation. **Christophe Hirtz:** Writing – review & editing, Writing – original draft, Supervision, Resources, Project administration, Methodology, Investigation, Funding acquisition, Conceptualization. **Isabelle Carriere:** Writing – original draft, Visualization, Software, Methodology, Investigation, Formal analysis, Data curation. **Karim Bennys:** Writing – review & editing, Supervision, Resources. **Laure-Anne Gutierrez:** Writing – review & editing, Writing – original draft, Visualization, Validation, Software, Methodology, Investigation, Formal analysis, Data curation. **Jana Kindermans:** Writing – review & editing, Methodology, Investigation. **Catherine Helmer:** Writing – review & editing, Supervision, Resources, Project administration, Investigation. **Audrey Gabelle:** Writing – review & editing, Writing – original draft, Supervision, Investigation, Funding acquisition, Conceptualization. **Sylvain Lehmann:** Writing – review & editing, Writing – original draft, Supervision, Resources, Project administration, Methodology, Investigation, Funding acquisition, Conceptualization. **Claudine Berr:** Writing – review & editing, Writing – original draft, Supervision, Software, Resources, Project administration, Methodology, Investigation, Funding acquisition, Formal analysis, Conceptualization.

## Declaration of competing interest

The authors declare that they have no known competing financial interests or personal relationships that could have appeared to influence the work reported in this paper.

## Appendix Supplementary materials

Image, application 1
