# Supplementary material for: A six-year risk assessment for dementia and Alzheimer's disease in the general population through immunoprecipitation-mass spectrometry plasma amyloid quantification
Source: J Prev Alzheimers Dis. 2025 Apr 19;12(7):100186. doi: 10.1016/j.tjpad.2025.100186 (PMC12321636; doi:10.1016/j.tjpad.2025.100186)
Supplement: Supplementary file 1 [file mmc1.docx]

**Supplemental Table S1: Association of plasma biomarkers with Alzheimer dementia risk***

| Plasma Biomarkers |  |  | Model 1 | | Model 2 | |
| --- | --- | --- | --- | --- | --- | --- |
|  |  | N | HR [95%CI] | *P*-value | HR [95%CI] | *P*-value |
| Aβ40 (pg/mL) | T1: ≤7.76 | 104 | 1 |  | 1 |  |
|  | T2: ]7.76-9.48] | 98 | **2.16 [1.11-4.24]** | **0.02** | **2.61 [1.18-5.77]** | **0.02** |
|  | T3: >9.48 | 98 | 1.67 [0.80-3.46] | 0.17 | **2.60 [1.06-6.36]** | **0.04** |
| Aβ42 (pg/mL) | T1: ≤0.33 | 100 | 1.56 [0.76-3.22] | 0.23 | 1.29 [0.53-3.13] | 0.57 |
|  | T2: ]0.33-0.41] | 96 | **1.98 [1.03-3.81]** | **0.04** | 2.05 [0.98-4.28] | 0.06 |
|  | T3:>0.41 | 104 | 1 |  | 1 |  |
| Composite biomarker | T1: ≤ -0.009 | 104 | 1 |  | 1 |  |
|  | T2: ]-0.009-0.62] | 102 | 1.09 [0.55-2.18] | 0.81 | 1.22 [0.54-2.75] | 0.63 |
|  | T3: >0.62 | 94 | **2.97 [1.47-6.01]** | **0.002** | **3.62 [1.61-8.16]** | **0.002** |
| Aβ42/Aβ40 Ratio | T1: ≤0.039 | 95 | **2.15 [1.08-4.27]** | **0.03** | **2.88 [1.27-6.51]** | **0.01** |
|  | T2: ]0.039-0.045] | 100 | 1.97 [0.93-4.19] | 0.08 | 1.99 [0.85-4.65] | 0.11 |
|  | T3: >0.045 | 105 | 1 |  | 1 |  |

The reference tertile is the lowest for Aβ40 and the composite biomarker and the highest tertile for Aβ42 and Aβ42/Aβ40 ratio.

Model 1 is adjusted for age, age^2^, center, gender and education.

Model 2: model 1 further adjusted for BMI, estimated glomerular filtration rate (eGFR), diabetes, cardiovascular pathologies, hypertension, living alone and ApoE4 genotype and with multiple imputations for added covariates.

HR= Hazard Ratios

*Analyses performed on 300 subjects including 84 AD cases.

# Supplemental Table S2: Association of plasma biomarkers with all-type dementia risk in participants with eGFR ≥60 mL/min/1.73m^2^, N=209

|  |  |  | Model 1 | | Model 2a | | Model 2b | |
| --- | --- | --- | --- | --- | --- | --- | --- | --- |
| Biomarkers |  | N | HR [95%CI] | *P*-value | HR [95%CI] | *P*-value | HR [95%CI] | *P*-value |
|  | T1: ≤7.76 | 87 | 1 |  | 1 |  | 1 |  |
| Aβ40 (pg/mL) | T2: ]7.76-9.48] | 72 | **2.49 [1.14-5.46]** | **0.02** | **3.29 [1.22-8.89]** | **0.02** | **3.52 [1.27-9.73]** | **0.02** |
|  | T3: >9.48 | 50 | 1.10 [0.45-2.69] | 0.83 | 1.57 [0.58-4.21] | 0.37 | 2.29 [0.74-7.1] | 0.15 |
|  | T1: ≤0.33 | 82 | **3.19 [1.30-7.81]** | **0.01** | **4.70 [1.33-16.68]** | **0.02** | **4.20 [1.18-15.02]** | **0.03** |
| Aβ42 (pg/mL) | T2: ]0.33-0.41] | 76 | **3.04 [1.35-6.88]** | **0.008** | **4.30 [1.58-11.69]** | **0.004** | **3.99 [1.46-10.93]** | **0.007** |
|  | T3:>0.41 | 51 | 1 |  | 1 |  | 1 |  |
|  | T1: ≤ -0.009 | 74 | 1 |  | 1 |  | 1 |  |
| Composite biomarker | T2: ]-0.009-0.62] | 73 | 1.02 [0.49-2.14] | 0.95 | 1.34 [0.48-3.72] | 0.58 | 1.55 [0.51-4.70] | 0.44 |
|  | T3: >0.62 | 62 | **4.08 [1.75-9.51]** | **0.001** | **8.02 [2.45-26.25]** | **0.0006** | **8.65 [2.51-29.79]** | **0.0006** |
| Aβ42/Aβ40 Ratio | T1: ≤0.039 | 61 | **2.59 [1.13-5.90]** | **0.02** | **4.87 [1.62-14.62]** | **0.005** | **7.00 [1.96-25.02]** | **0.003** |
|  | T2: ]0.039-0.045] | 75 | 2.16 [0.96-4.83] | 0.06 | **3.14 [1.02-9.65]** | **0.05** | **3.80 [1.07-13.53]** | **0.04** |
|  | T3: >0.045 | 73 | 1 |  | 1 |  | 1 |  |

Model 1 adjusted for age, age^2^, center, gender and education

Model 2a: Model 1 further adjusted for BMI, diabetes, cardiovascular pathologies, hypertension, living alone and ApoE4 genotype and with multiple imputations for added covariates.

Model 2b: Model 1 further adjusted for BMI, estimated glomerular filtration rate, diabetes, cardiovascular pathologies, hypertension, living alone and ApoE4 genotype and with multiple imputations for added covariates.

133 subcohort non-cases, 17 subcohort cases and 59 non-subcohort cases.

# Supplemental Table S3: Correlation between amyloid biomarkers and eGFR, n=324

|  | **Aβ40** | **Aβ42** | **APP669-711**  **(Aβ3-40)** | **Aβ42/Aβ40 ratio** | **Composite biomarker** |
| --- | --- | --- | --- | --- | --- |
| **Spearman correlation coefficients** | -0.46 | -0.35 | -0.39 | 0.12 | -0.08 |
| **Prob > \|r\| under H0: Rho=0** | **<0.0001** | **<0.0001** | **<0.0001** | **0.04** | 0.16 |
